# A Standardized Culture Medium for Comparative Drug Efficacy Evaluation Across *Plasmodium* and *Babesia* Species

**DOI:** 10.21769/BioProtoc.5625

**Published:** 2026-03-05

**Authors:** Pratap Vydyam, Choukri Ben Mamoun

**Affiliations:** Department of Internal Medicine (Section of Infectious Diseases), Microbial Pathogenesis, and Pathology, Yale School of Medicine, New Haven, CT, USA

**Keywords:** Human malaria, Human babesiosis, *Babesia*, Plasmodium, DFS20, Drug screening, antifolates, Drug resistance, Protozoan parasites

## Abstract

The discovery of broad-spectrum antiparasitic agents relies on the ability to evaluate drug efficacy under harmonized in vitro conditions across related species. However, current drug screening pipelines for intraerythrocytic parasites are constrained by the use of species-specific media with distinct nutrient compositions and serum sources, which hinder direct comparison of compound potency. To address this gap, we describe a unified erythrocytic culture system based on DMEM/F12 supplemented with 20% fetal bovine serum (DFS20), which supports robust asexual growth of multiple *Plasmodium falciparum* strains (3D7, Dd2, HB3, V1/S), *Babesia duncani, Babesia divergens* (Rouen 87), and *Babesia MO1*. Parasite proliferation and morphology in DFS20 are comparable to those observed in established species-specific media such as RPMI-1640 for *P. falciparum* and *B. divergens* and HL-1/Claycomb/DMEM/F12/SFM for *B. duncani*, while eliminating reliance on undefined or discontinued proprietary components. Importantly, this standardized medium enables cross-species growth inhibition assays for direct comparison of drug efficacy under identical conditions. Using this platform, we recently screened dihydrotriazines and biguanides targeting the conserved DHFR-TS enzymes and identified potent antifolate candidates with broad-spectrum activity against *Babesia* and *Plasmodium* species. For *B. duncani*, which is uniquely supported by both a continuous in vitro human erythrocyte culture system and a lethal in vivo mouse infection model, integration with the in-culture and in-mouse (ICIM) pipeline enables systematic validation of pharmacodynamics, pharmacokinetics, resistance, and toxicity. This unified DFS20-based system establishes a scalable and reproducible protocol for harmonized drug efficacy evaluation across intraerythrocytic parasites and provides a foundation for the development and prioritization of pan-antiparasitic therapies.

Key features

• Common culture medium (CCM): DFS20 supports consistent *Babesia* and *Plasmodium* growth, enabling standardized, reproducible drug screening across intraerythrocytic protozoan parasites.

• Scalable antiparasitic drug screening: SYBR Green I–based in vitro assay adaptable to low-, medium-, or high-throughput compound testing in both drug-sensitive and drug-resistant strains.

• Unified red blood cell–based platform that may accelerate efforts to culture challenging or unculturable parasites, thereby expanding opportunities for drug discovery and mechanistic studies.

## Background

The discovery of broad-spectrum antiparasitic agents requires in vitro systems that allow direct comparison of compound efficacy across multiple species under uniform conditions. However, existing intraerythrocytic parasite culture platforms rely on species-specific media with differing nutrient compositions, serum sources, and undefined protein content. These inconsistencies affect parasite growth dynamics and drug potency measurements (e.g., IC_50_ values), making it difficult to determine whether observed efficacy differences are due to true biological variation or culture-dependent artifacts. As a result, cross-species prioritization of chemical scaffolds with pan-apicomplexan potential remains challenging.

The clinical and epidemiological burden of intraerythrocytic apicomplexan parasites underscores the importance of developing such comparative approaches. According to the *World Malaria Report 2024*, there were an estimated 263 million malaria cases and approximately 597,000 deaths in 2023, with 94% of cases and 95% of deaths occurring in the WHO African Region [1]. Children under five accounted for approximately 76% of all malaria deaths, highlighting their disproportionate vulnerability [1]. Human babesiosis, caused by tick-transmitted *Babesia* species such as *B. microti, B. duncani, B. divergens*, and *B. MO1*, is an emerging zoonosis with increasing incidence in North America, Europe, and Asia [2–7]. According to the Centers for Disease Control and Prevention (CDC), tick-borne apicomplexan parasitic diseases have increased globally, with over 2,000 annual cases in the United States alone [8]. Resistance to first-line therapies has been documented in both genera: in *Plasmodium*, through mutations in *PfCRT, DHFR, DHPS*, and *ATP4* [9–13], and in *Babesia*, notably in *CytB* and ribosomal proteins [14–17]. These shared trends of rising incidence and resistance highlight the need for new broad-spectrum antiparasitic drugs and standardized platforms that enable cross-species drug efficacy evaluation under harmonized conditions.

**Figure 1.**
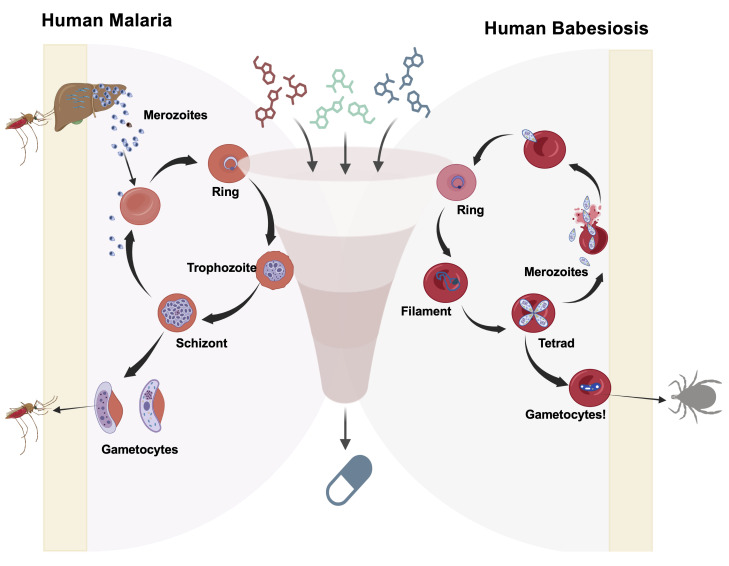
Schematic representation of a high-throughput screening workflow used to evaluate pan-antiparasitic drugs targeting the in vitro asexual life cycle stages of *Plasmodium* and *Babesia* parasites. The diagram illustrates the major intraerythrocytic developmental stages [ring, trophozoite, and schizont for *Plasmodium falciparum* (3D7, Dd2, HB1, and V1/S); ring, filament, and tetrad stages for *Babesia (B. duncani, B. divergens Rouen87*, and *B. MO1*)] and depicts merozoite invasion into fresh human red blood cells (RBCs) and continuous asexual replication. Additionally, the figure highlights the potential development of gametocytes in both parasites. A screening funnel illustrates the evaluation of diverse candidate compounds across the erythrocytic stages of malaria and babesiosis parasites.

*Plasmodium* and *Babesia* species share key biological and pharmacological features that make cross-species drug development viable. They are obligate intraerythrocytic parasites that invade host erythrocytes using specialized cellular machineries, undergo clonal asexual replication (schizogony in *Plasmodium* and binary fission in *Babesia*), and depend on conserved metabolic pathways localized in essential organelles such as the apicoplast and mitochondrion [18–23] (Figure 1). The single mitochondrion is indispensable in both genera and serves as a validated pan-apicomplexan drug target. Mitochondrial *bc_1_* complex inhibitors such as atovaquone and endochin-like quinolones (ELQs) exhibit potent activity against both *Plasmodium* and *Babesia* species [14,22–27]. Resistance to these agents has been linked to conserved mutations in the cytochrome b (*CytB*) gene in both genera, confirming shared druggable vulnerabilities and resistance trajectories [14, 17,26,28–31]. Likewise, conserved enzymes such as DHFR-TS and core mitochondrial metabolic enzymes represent shared vulnerabilities that have been pharmacologically targeted in both parasites using antifolate and glycolytic inhibitor chemotypes [18,26,32–35]. These and additional shared, yet underexplored, vulnerabilities highlight the need for a unified in vitro system for drug screening and resistance testing (Figure 1).

Despite these shared vulnerabilities, current culture methodologies are highly fragmented. *P. falciparum* and *B. divergens* are typically cultured in RPMI-1640 supplemented with human serum or lipid-based substitutes such as Albumax [36,37]. In contrast, the first in vitro culture assays for *B. duncani* relied exclusively on HL-1 and Claycomb media [22,26,38], both of which are proprietary and inconsistently available. A major advance occurred in 2021, when *B. duncani* was shown to grow continuously in human erythrocytes using DMEM/F-12 supplemented with 20% fetal bovine serum (DFS20) [39,40], providing a defined and widely accessible alternative to these media. Although *B. MO1* displays growth characteristics similar to *B. divergens* [34], it has not yet been incorporated into a unified culture system. Of particular importance, *B. duncani* remains the only *Babesia* species for which both continuous in vitro culture in human erythrocytes and a lethal in vivo murine infection model are available [28,39–41], making it uniquely suited for translational validation of drug candidates.

To address these limitations, we evaluated DMEM/F-12 supplemented with 20% fetal bovine serum (DFS20) as a common culture medium (CCM) capable of supporting the growth of *Plasmodium* and *Babesia*. We demonstrate that DFS20 supports robust asexual replication of *P. falciparum* (3D7, Dd2, HB3, V1/S), *B. duncani, B. divergens (Rouen 87*), and *B. MO1* with growth kinetics and morphology comparable to those observed in their respective traditional media [33–35]. In addition, we emphasize that DFS20 functions as a unified and practical solution for comparative drug evaluation by eliminating media-dependent variability across species. This enables consistent readouts, improves assay reproducibility, and positions DFS20 as a scalable platform for pan-apicomplexan drug screening. This unified culture system enables standardized, cross-species drug susceptibility testing and facilitates screening of compounds targeting conserved metabolic vulnerabilities. Furthermore, when integrated with the in-culture and in-mouse (ICIM) model, currently applicable only to *B. duncani*, it serves as a proof-of-concept for how this standardized system could support parallel assessment of pharmacodynamics, pharmacokinetics, toxicity, and resistance emergence. By replacing fragmented media systems with a single, scalable, and reproducible platform, this protocol establishes a foundation for cross-species efficacy assessment, resistance profiling, and the identification and prioritization of next-generation pan-apicomplexan therapeutics.

## Materials and reagents

**Biological materials**

1. Human packed RBCs: Human erythrocytes (Group type A+ for optimal growth) obtained from American Red Cross or Interstate Blood Bank-USA, collected in citrate phosphate dextrose (CPD), washed twice with incomplete medium, and stored at 4 °C for a maximum of 4 weeks

2. Parasite species and strains:

a. *Plasmodium falciparum 3D7:* (MRA-102; BEI Resources)

b. *Plasmodium falciparum Dd2:* (MRA-150; BEI Resources)

c. *Plasmodium falciparum HB3:* (MRA-1227; BEI Resources)

d. *Babesia duncani WA1:* (NR-12311; BEI Resources)

e. *Babesia divergens Rouen87:* (NR-52008; BEI Resources)

f. *Babesia MO1:* (NR-50441; BEI Resources)

**Reagents**

1. RPMI-1640 (Gibco, catalog number: 11875093); store at 4 °C

2. Albumax-II (Invitrogen, catalog number: 11021045); store at 4 °C

3. L-Glutamine (Gibco, catalog number: 25030-081); store at -20 °C

4. Hypoxanthine (Sigma, catalog number: H9636)

5. HEPES (Gibco, catalog number: 15630080); store at 4 °C

6. Sodium bicarbonate (NaHCO_3_) (Sigma, catalog number: S5761); store at 4 °C

7. Gentamicin (Gibco, catalog number: 15710-072)

8. DMEM-F12 (Gibco, catalog number: 11320033); store at 4 °C

9. Heat-inactivated fetal bovine serum (FBS) (Gibco, catalog number: 16140071); store at -80 °C

10. 50× HT Media Supplement Hybri-Max^TM^ (Sigma, catalog number: H0137); store at -20 °C

11. 100× Penicillin/Streptomycin (Gibco, catalog number: 15240-062); store at -20 °C

12. 0.008% Saponin (Sigma, catalog number: S7900)

13. 0.08% Triton X-100 (Sigma, catalog number: T8787)

14. 20 mM Tris-HCl, pH 7.5 (Sigma, catalog number: T2194)

15. 5 mM EDTA (Sigma, catalog number: E6758)

16. SYBR Green-I (Invitrogen, catalog number: S7563), diluted to 0.01% in lysis buffer; protect SYBR Green-I from light to prevent photobleaching and store at -20 °C

17. Dimethyl sulfoxide (DMSO) (Sigma, catalog number: D2650)

18. Phosphate-buffered saline (PBS) (Gibco, catalog number: 10010023)

19. Giemsa stain solution 1 (three-step fixative) (Fisher Scientific, catalog number: 22050273)

20. Giemsa stain solution 2 (three-step solution I) (Fisher Scientific, catalog number: 22050274)

21. Giemsa stain solution 3 (three-step solution II) (Fisher Scientific, catalog number: 22050275)

22. Antifolate drugs: Dihydrotriazines (51 compounds) and biguanides (28 compounds), sourced from Jacobus Pharmaceuticals, purity ≥95% (confirmed by reverse-phase HPLC)

**Solutions**

1. RPMI complete culture medium (see Recipes)

2. DFS20 complete culture medium (see Recipes)

3. SYBR Green-I lysis buffer (see Recipes)

**Recipes**

**1. RPMI complete culture medium**

**Table:** 

Reagent	Final concentration
RPMI-1640	99.3%
Albumax-II	0.5%
L-Glutamine	2 mM
Hypoxanthine	50 mg/L
NaHCO_3_	0.225%
HEPES	25 mM
Gentamicin	10 mg/mL

**2. DFS20 complete culture medium**

**Table:** 

Reagent	Final concentration
DMEM-F12	77%
Heat-inactivated FBS	20%
50× HT Media Supplement Hybri-Max^TM^	2%
L-Glutamine	1%
Penicillin/Streptomycin	1%
Gentamicin	1%

**3. SYBR Green-I lysis buffer**

**Table:** 

Reagent	Final concentration
Saponin	0.008%
Triton X-100	0.08%
Tris-HCl, pH 7.5	20 mM
EDTA	5 mM
SYBR Green-I	0.01%

*Note: Prepare the lysis buffer in distilled water and add SYBR Green-I to a concentration of 0.01%. Protect SYBR Green-I from light to prevent photobleaching.*

## Equipment

1. Biological safety cabinet (Sterile GARD III), Advance biological safety cabinet (The Baker Company, catalog number: SG603)

2. CO_2_ incubator (Thermo Scientific, model Heracell VIOS 160i), set to 37 °C, 5% CO_2_, 2% O_2_, 93% N_2_ for *Babesia* spp. and 4% O_2_, 5% CO_2_, 91% N_2_ for *P. falciparum*

3. Sorvall legend XTR centrifuge (Thermo Scientific, catalog number: 75004521) with rotor for 15 and 50 mL tubes

4. Microscope (Nikon, model: Eclipse 5Oi)

5. Water bath (Fisher brand, model: FSGPD15D) Precision GP 10, set to 37 °C

6. Ultrasonic Bath (Branson Bransonic^®^ M Mechanical Bath, model: M2800)

7. 96-well flat clear-bottom tissue culture plates (Corning, catalog number: 3596)

8. Greiner Bio-One Cellstar 96-well black flat bottom (Thermo Scientific, catalog number: 237108)

9. Multichannel pipettor (Eppendorf, Research Plus, 10–200 μL and 5–50 μL)

10. Microplate reader: BioTek Synergy Mx, with 480 nm excitation and 540 nm emission filters (top reading mode)

11. Vortex mixer (Scientific Industries, model: Vortex-Genie 2)

12. Sterile 15 and 50 mL conical tubes (Falcon, catalog numbers: 352096 and 352070)

13. Sterile pipette tips and pipettors (10 μL, 200 μL, 1,000 μL)

14. Sterile 1.5 mL microcentrifuge tubes (Eppendorf, catalog number: 022431081)

## Software and datasets

1. Microsoft Excel, for data organization and initial calculations

2. GraphPad Prism (version 9.1.2 or advanced), for data analysis, plotting the heat maps, and sigmoidal (S) inhibition curves for IC_50_ determination with standard deviation (SD)

## Procedure

**Part I. Preparation of parasite cultures**

Time required: 2–3 days for culture establishment and synchronization.

**A. Prepare erythrocytes**

1. Centrifuge A+ packed RBCs at 1,000 rcf (acceleration 9 and break 0) for 15 min at 4 °C to separate the remaining buffy coat and RBCs.

2. Wash RBCs three times with 10 mL of cold 1× PBS (centrifuge at 700 rcf for 5 min each wash).

3. Resuspend RBCs in incomplete RPMI-1640 medium to achieve 50% hematocrit (e.g., 10 mL of packed RBCs + 10 mL of DFS20) and store at 4 °C.

**B. Determining initial parasitemia**

1. Prepare thin blood smears from stock cultures of *Babesia* spp. and *Plasmodium* spp. strains, fix with a fixative solution (10 s), stain with Giemsa (Solution I and II) for 30 s each, wash off excess stain, and air-dry the slide.

2. Count at least 3,000 RBCs under a 100× oil immersion objective across 10 different fields on the slide to calculate the percentage of parasitemia.

3. Dilute cultures with fresh RBCs in complete DFS20 and/or RPMI-1640 medium to achieve 0.2% parasitemia at 5% hematocrit for assay setup.

4. Calculate in vitro parasitemia count as instructed in [33,35,42].

**C. Maintain stock cultures**

1. Hematocrit: Hematocrit (HCT) refers to the percentage of RBCs in a given volume of blood, representing the packed cell volume. Fresh human RBCs are stored at 50% hematocrit for laboratory use and maintained at 4 °C for up to two weeks. Routine asexual blood-stage cultures are maintained at 2%–5% hematocrit, depending on growth requirements.

2. Percent parasitemia: Parasitemia refers to the total number of all stages of (mixed stages) parasite-infected RBCs within a total red blood cell population in the in vitro cultures and is commonly expressed as a percentage. Routine maintenance cultures are kept at 1%–3% parasitemia to prevent overgrowth and nutrient limitation. Cultures that reach >5% parasitemia are diluted with fresh RBCs and complete medium to maintain healthy exponential growth and prevent synchronization loss.

3. *Babesia duncani (WA-1), B. divergens (Rouen87)*, and *B. MO1*: Culture parasites in DFS20 medium at 5% hematocrit (A^+^ human RBCs) at 37 °C under a gas mixture of 2% O_2_, 5% CO_2_, and 93% N_2_. Change medium daily and determine parasitemia from Giemsa-stained thin blood smears by light microscopy.

4. *Plasmodium falciparum (3D7, Dd2, HB3)* strains: Culture parasites in RPMI-1640 complete culture medium at 3%–5% hematocrit (A^+^ human RBCs) at 37 °C under a gas mixture of 4% O_2_, 5% CO_2_, and 91% N_2_. Change medium daily and monitor parasitemia as above.

5. Subculture parasites every two generations and maintain parasitemia between 1% and 4% to ensure optimal growth conditions. For *Plasmodium* strains, synchronize cultures every 5–8 generations.

**Critical:**

1. Ensure that stock cultures are healthy before starting the assay. Monitor parasite growth to confirm exponential growth. *B. duncani* parasitemia typically doubles every 24 h, while that of *P. falciparum* increases by 4-fold every 48 h (one generation time is 48 h).

2. Include all three antibiotics when culturing multiple parasite species in parallel to minimize contamination risk, although gentamicin alone is sufficient for cultures.

**Part II. Preparation of drug solutions**

Time required: 1–2 h.

**A. Prepare stock solutions**

1. Dissolve compounds in 100% DMSO to make 10 mM stock solutions.

*Note: Ensure complete dissolution by vortexing for 10–15 s. Store at -20 °C for long-term storage.*

**Troubleshooting:** If precipitation occurs, warm to 50 °C and vortex again. Use an ultrasonic bath if necessary.

**B. Prepare working dilutions**

1. Perform serial 2-fold dilutions in complete DFS20 medium to create a 4-step or 12-step concentration range (e.g., 100, 50, 10, and 5 nM to 0.049 nM final concentration in assay wells).

2. Use a 96-well clear flat-bottom plate for dilutions to facilitate multichannel pipetting.

3. Ensure final DMSO concentration in assay wells does not exceed 0.5% (v/v).

4. Prepare working drug dilutions at 2× concentrations.

*Note: Prepare fresh dilutions on the day of the experiment to avoid degradation or precipitation.*

**Part III. Set up the growth inhibition assay**

Time required: 1–2 h for setup.

**A. Prepare assay plates in DFS20 medium**

**Figure 2.**
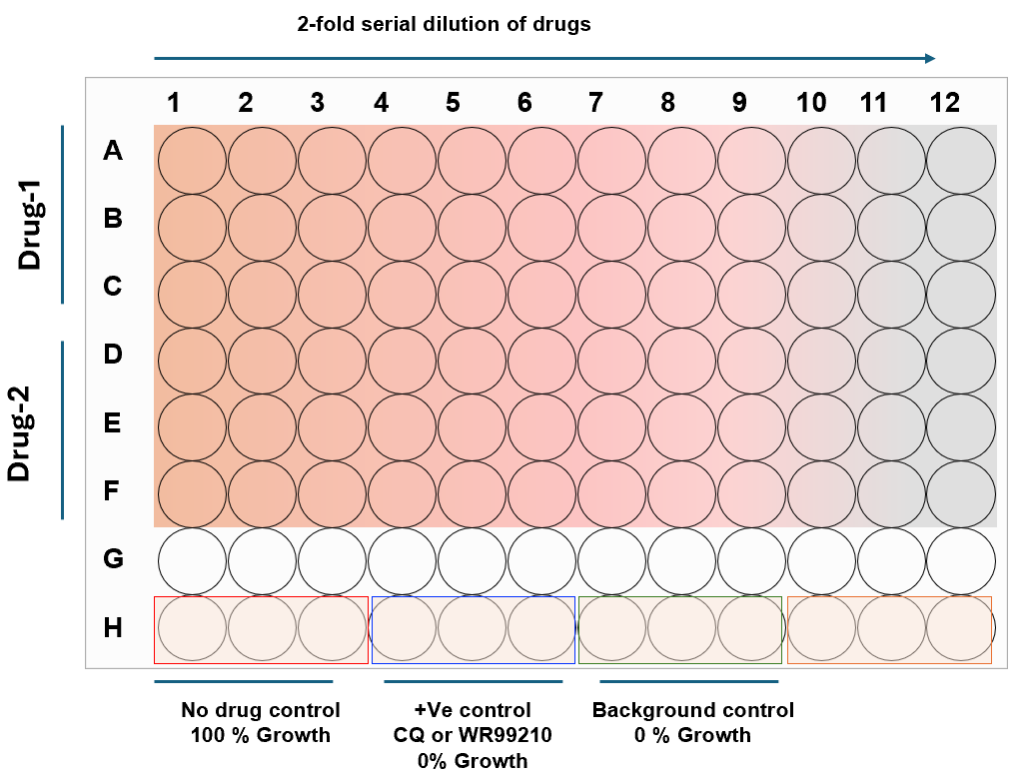
96-well assay plate showing a schematic representation of serial dilutions of drugs, including positive and negative controls. Each well accommodates 200 μL of culture volume (*Plasmodium* or *Babesia* parasites) with drugs at various concentrations, prepared by 2-fold serial dilutions. Gradient color indicates the concentration differences in the 96-well flat clear-bottom plate.

**Figure 3.**
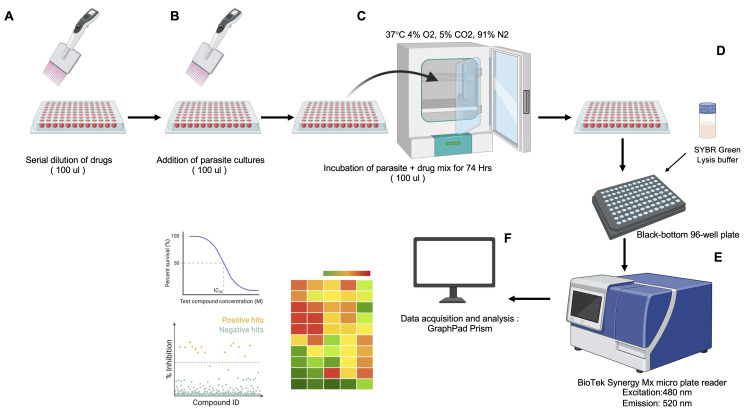
Diagram of the SYBR Green I–based high-throughput screening (HTS) assay for assessing drug efficacy against the in vitro asexual life cycle stages of *Plasmodium* and *Babesia* parasites in human red blood cells (hRBCs). (A) Preparation of a 96-well clear flat-bottom drug plate with 2-fold serial dilutions in 100 μL of DFS20 medium. (B) Addition of 100 μL of hRBC-infected parasite culture to the drug plate (96-well flat clear bottom plate) using a multichannel pipette. (C) Incubation of the parasite–drug mixture in a 96-well clear flat-bottom plate at 37 °C in a chamber with 4% O_2_, 5% CO_2_, 91% N_2_, and humidification for 72 h. (D) Transfer of incubated cultures to a black-bottom 96-well plate, followed by the addition of an equal volume of SYBR Green I lysis buffer to the parasite culture. (E) Measurement of SYBR Green I fluorescence to quantify parasite DNA content, with excitation at 480 nm and emission at 520 nm. (F) Export of fluorescence data for analysis, including generation of heat maps and determination of IC_50_ with standard deviation values using Microsoft Excel and GraphPad Prism.

1. In a 96-well flat-clear bottom plate (columns 1–12, rows A–H), add 100 μL of DFS20 medium to all wells except those designated for the initial drug dilutions (e.g., first column of triplicate wells for each drug, such as A1, B1, C1) (see Figure 2).

2. Add 200 μL of each drug dilution (2× working concentration, e.g., 200 nM for a 100 nM final working concentration) to the first column of test wells in triplicate (e.g., rows A–F for two different drugs; see Figure 3).

3. Perform a 2-fold serial dilution by transferring 100 μL from the first wells (2× drug concentration) to the subsequent wells in each row, continuing until the 12th well. Discard the final 100 μL from the 12th well to maintain consistent volumes.

4. Add 100 μL of DFS20 medium with 0.5% DMSO to no-drug control wells (maximum parasite growth; H1–H3).

5. Add 100 μL of DFS20 medium with 2 μM WR99210 (for *Babesia* spp.) or 10× IC_50 _concentration of chloroquine (for *Plasmodium* spp.) to positive control wells (for 100% parasite inhibition; wells H4–H6).

*Note: Ensure a uniform cell suspension by gently pipetting before dispensing. From this point forward, all parasite cultures and drug treatments are maintained in DFS20 medium.*

**B. Add parasite cultures to the assay plate**

1. Add 100 μL of parasite culture (0.4% parasitemia, 10% hematocrit in DFS20 medium, 2× concentration) to all test wells with 2× drug concentrations. The final volume in each well should be 200 μL, with both drug and parasite at 1× concentrations.

2. Add 100 μL of uninfected RBCs (10% hematocrit in DFS20 medium) to background control wells (H10–H12).

**Critical:** *Plasmodium* cultures grown in RPMI-1640 need to be washed twice with DFS20 (700 rcf, 5 min, at room temperature). Use a multichannel pipette for consistency. Pipette slowly to avoid bubbles.

**C. Incubate plates**

1. Cover the plate with a low-evaporation lid to allow gas exchange.

2. Incubate at 37 °C in a humidified chamber with 4% O_2_, 5% CO_2_, and 91% N_2_ for 72 h.

**D. Measurement of parasite growth**

Time required: 2–3 h.

1. Prepare SYBR Green-I lysis buffer according to the provided recipe.

2. After 72 h of incubation, remove the 96-well clear flat-bottom drug and parasite mix plate from the incubator.

3. Gently resuspend the culture in each well by pipetting up and down 5 times to ensure homogeneity.

4. Transfer 100 μL of each culture to a new black flat-bottom 96-well plate containing 100 μL of SYBR Green-I lysis buffer per well.

5. Mix gently by pipetting and incubate the plate at 37 °C in the dark (covered with aluminum foil) for 15 min to allow complete lysis and DNA staining.

6. Read fluorescence using a BioTek Synergy Mx Microplate Reader (or equivalent, e.g., Spectra Mx) with excitation at 480 nm and emission at 540 nm. (These wavelengths were routinely used and well-established to detect the SYBR Green base assessment.)

**Critical:** Protect SYBR Green-I from light to prevent photobleaching. Some protocols may require a 1-h incubation for complete lysis; adjust as per your experimental requirements.

**Troubleshooting:** If fluorescence is low, ensure thorough mixing for complete RBC lysis.

**E. Data analysis**

Time required: 1–2 h.

1. After completing the fluorescence reading, export the total fluorescence values from the assay to an Excel spreadsheet.

2. Each assay plate includes positive control wells (e.g., treated with WR99210 or chloroquine, CQ) and background control wells (uninfected RBCs).

3. Calculate the average fluorescence (AFU) of the uninfected RBC wells (background control). Subtract this background fluorescence value from all test wells, negative control wells (0.1% DMSO), and positive control wells to normalize the data.

4. For each test well, calculate the percent growth and inhibition using the following formulas:

$$\%\;Growth=\frac{\left(Fluorescence\;of\;test\;well-Fluorescence\;of\;positive\;control\right)}{\left(Fluorescence\;of\;negative\;control-\;Fluorescence\;of\;positive\;control\right)}\times 100$$

% Inhibition=100-% Growth

5. Export the % inhibition values of each drug concentration in the group format in GraphPad Prism and adjust the color format. Generate the heat maps.

6. Plot percent inhibition (y-axis) against log-transformed drug concentrations (x-axis) using GraphPad Prism.

7. Fit a sigmoidal dose-response curve (variable slope) to calculate IC_50_ values with derived SD from biological triplicate experiments

8. Perform the analysis using data from three independent experiments, each with technical triplicates.

**Critical:**

1. Negative control (0.1% DMSO) represents 0% inhibition (maximum parasite growth).

2. Positive control (WR99210 or CQ-treated) represents 100% inhibition (no parasite growth).

3. Uninfected RBC wells serve as the background control for fluorescence subtraction.

**Troubleshooting:** If IC_50_ curves appear irregular, verify pipetting errors or insufficient drug solubility. Remove or adjust obvious outliers from the dataset as needed.

## Validation of protocol

This protocol (or parts of it) has been used and validated in the following research article:

- Vydyam et al. [33]. In vitro efficacy of next-generation dihydrotriazines and biguanides against babesiosis and malaria parasites. *Antimicrob Agents Chemother* (Figure 4).

## General notes and troubleshooting

1. Standardized culture medium (DFS20): Using DFS20 ensures consistent nutrient availability (putrescine, linoleic acid, lipoic acid) across *Babesia* and *P. falciparum* cultures, critical for cross-species comparisons.

2. Accurate parasitemia adjustment: Starting at 0.2% parasitemia ensures logarithmic growth during the 72-h incubation, maximizing assay sensitivity.

3. SYBR Green-I fluorescence: Thorough mixing during lysis is essential for consistent fluorescence readings. Avoid bubbles to prevent reader artifacts.

4. Once a reproducible culture system for *B. microti* becomes available, we anticipate that this platform can be extended to include this species and additional *Babesia* parasites in future studies.

**Personal notes and tips**

1. Culture stability: Monitor cultures daily. If parasitemia exceeds 5%, subculture to avoid stress-induced variability in drug response.

2. Compound solubility: Some compounds may exhibit limited solubility. Sonicate briefly or warm stock solutions to 37 °C to ensure complete dissolution.

3. Pipetting accuracy: Use calibrated pipettes and practice consistent technique to minimize variability in drug dilutions and culture dispensing.
